# Analysis of bilevel positive airway pressure therapy in children based on weight

**DOI:** 10.1186/cc12079

**Published:** 2013-03-19

**Authors:** A Williams, T Abramo

**Affiliations:** 1 Vanderbilt University Medical Center, Nashville, TN, USA

## Introduction

Obesity as a comorbidity adds a challenge when treating children presenting to the PED in status asthmaticus. Bilevel positive airway pressure (BiPAP) is an accepted treatment modality for children in status asthmaticus. Our purpose was to analyze BiPAP use and outcomes for children with status asthmaticus and obesity in our PED.

## Methods

Patients placed on BiPAP in the PED for status asthmaticus from 1 January 2010 to 31 August 2012 were included in the analysis. Subjects were divided into moderate and severe exacerbations and then further subdivided into the following growth curve-based weight subgroups: <90 percentile, 90 to 97 percentile and >97 percentile. Subjects received standard asthma therapies in addition to BiPAP. Data were obtained at the bedside by the respiratory therapist or collected retrospectively by study investigators. Data were stored and analyzed using a RedCap database.

## Results

Three hundred and fifty-nine subjects were analyzed. Table 1 shows the time on BiPAP per visit. Children whose weight was >97 percentile revealed trends towards longer treatment times on BiPAP compared with the other two groups. The moderate subjects who weighed >97 percentile had statistically significant longer treatment periods (*P <*0.006) when compared with the <90 percentile moderate group. Initial BiPAP settings are listed in Table 2. When controlling for age, higher BiPAP settings correlated with increasing weight. There were no weight-based trends for admissions to the PICU or overall hospital lengths of stay. No serious complications were noted.

**Table 1:** Time on BiPAP by weight percentile and severity of asthma exacerbation (hours)

Moderate exacerbation				Severe exacerbation			Combined		
			
	<90	90 to 97	>97	<90	90 to 97	>97	<90	90 to 97	>97
	percentile	percentile	percentile	percentile	percentile	percentile	percentile	percentile	percentile
	(*n *= 113)	(*n *= 35)	(*n *= 36)	(*n *= 109)	(*n *= 35)	(*n *= 31)	(*n *= 222)	(*n *= 70)	(*n *= 67)
Mean	3.53	4.03	5.01	5.67	5.56	5.88	4.58	4.85	5.41
SD	3.35	3.24	4.26	4.78	4.03	7.25	4.25	3.75	5.81
Median	2.50	3.00	3.75	3.83	4.00	4.00	3.00	3.38	4.00

**Table 2:** Initial mean BiPAP settings by age and weight Percentile.

	<90 percentile		90 to 97 percentile		>97 percentile	
			
Age						
(years)	IPAP	EPAP	IPAP	EPAP	IPAP	EPAP
3 to 4	12.6 ± 2.4	6.7 ± 1.4	13.0 ± 2.2	6.5 ± 1.2	13.1 ± 1.9	6.7 ± 1.0
5 to 6	13.4 ± 2.4	6.9 ± 1.4	12.4 ± 2.0	6.6 ± 1.5	14.2 ± 1.3	7.0 ± 0.9
7 to 9	14.2 ± 2.2	7.4 ± 1.4	14.9 ± 2.7	7.7 ± 1.8	14.1 ± 2.4	7.1 ± 1.3
10 to 12	14.1 ± 2.7	7.3 ± 1.7	15.4 ± 4.1	7.9 ± 2.0	15.1 ± 3.1	8.0 ± 1.6
13 to 16	15.2 ± 4.2	8.6 ± 1.8	14.0 ± 1.8	7.7 ± 1.5	16.5 ± 3.9	8.8 ± 1.8

## Conclusion

This is the first study to analyze the weight effect on BiPAP application in children with status asthmaticus. Subjects who weighed more trended greater mean time on BiPAP and initial BiPAP settings. Weight did affect PICU admissions or overall length of hospital stay.

